# Early Mobilization Protocols in Critically Ill Pediatric Patients: A Scoping Review of Strategies, Tools and Perceived Barriers

**DOI:** 10.3390/children12050633

**Published:** 2025-05-14

**Authors:** Lizeth Dayana Noreña-Buitrón, Valeria Sanclemente-Cardoza, Maria Alejandra Espinosa-Cifuentes, Harold Andrés Payán-Salcedo, Jose Luis Estela-Zape

**Affiliations:** 1 Faculty of Health Sciences, Fundación Universitaria María Cano, Cali 760035, Colombia; lizethdayana752@gmail.com (L.D.N.-B.); valeriasanclemente0@gmail.com (V.S.-C.); 2 Faculty of Health, Universidad Santiago de Cali, Cali 760035, Colombia; malejandraec@gmail.com (M.A.E.-C.); haroldpayan00@usc.edu.co (H.A.P.-S.); 3 Health and Movement Research Group, Universidad Santiago de Cali, Cali 760035, Colombia

**Keywords:** early ambulation, pediatrics, guidelines as topic, critical care

## Abstract

Background/Objectives: We will describe the early mobilization protocols applied to critically ill pediatric patients in PICUs, analyzing the strategies employed, the tools used, and the barriers perceived by the healthcare team during their implementation. Methods: The scoping review followed the guidelines established by PRISMA-ScR. A search was conducted across five electronic databases: PubMed, Scopus, Web of Science, Dimensions AI, and ScienceDirect. Articles published in English that focused on pediatric patients aged 0 to 18 years were included. Results: A total of 3508 records were initially identified, of which 3422 articles were evaluated after duplicate removal. Subsequently, 12 studies that met the inclusion criteria were included. The methodological quality of the studies was mostly adequate, with 71.43% achieving scores between eight and nine on the Newcastle–Ottawa scale and 50% of the randomized clinical trials obtaining the maximum score of 7/7 on the Jadad scale. The interventions analyzed, including active bed mobility, bed cycling, and virtual reality, showed positive results in terms of feasibility and safety. The most frequently reported barriers to mobilization were hemodynamic instability, excessive sedation, pain, and lack of personnel and equipment. Conclusions: Early mobilization in pediatric PICUs is linked to improvements in mobility, reduced hospital stays, and shorter mechanical ventilation duration. However, its implementation is limited by barriers such as hemodynamic instability, excessive sedation, and lack of personnel and equipment. Further research is needed to establish uniform protocols, reduce these barriers, and optimize their effectiveness.

## 1. Introduction

Pediatric Intensive Care Units (PICUs) are high-complexity environments requiring specific therapeutic strategies for managing critically ill patients, which may limit physical activity and result in prolonged immobility [1,2]. Factors such as the use of analgesics, sedatives, and neuromuscular blockers to facilitate mechanical ventilation, reduce psychomotor agitation, and control pain are essential for maintaining respiratory stability [3,4]. However, these interventions may contribute to the development of Intensive Care Unit-acquired Weakness (ICUAW) [5,6], which is associated with prolonged hospital stays, difficulties in weaning from mechanical ventilation, increased infection risk, functional disability [7,8], and higher healthcare costs [9,10].

The incidence of ICUAW in pediatric populations is limited due to difficulties in establishing objective and reproducible muscle strength assessments in children, unlike the protocols available for adults [3,11]. Recent studies [8,12] report an incidence of 1.7% in pediatrics, notably lower than the 40% reported in adults; however, this figure may be an underestimate due to limited knowledge of the condition and challenges in clinical assessment in pediatrics [13,14,15]. This has resulted in a lack of data, limiting understanding of its incidence, progression, and prognosis [8,16].

**Figure 1:**
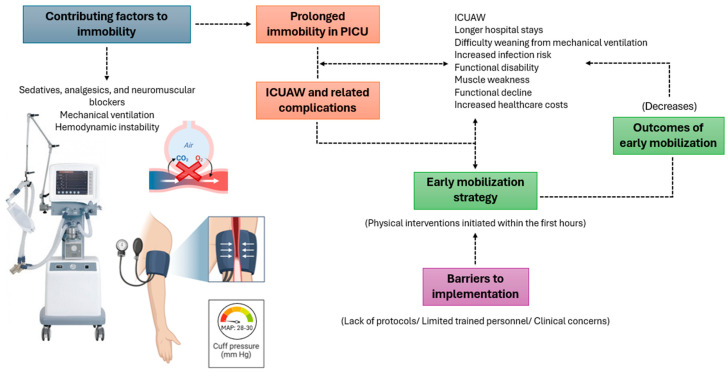
Contributing factors, barriers, and outcomes of early mobilization in PICU. PICU: Pediatric Intensive Care Units; ICUAW: Intensive Care Unit-acquired Weakness; MAP: Mean Arterial Pressure.

In recent years, despite declining mortality rates in Pediatric Intensive Care Units (PICUs), an increase in the prevalence of functional limitations at discharge has been documented in this population [17,18,19]. Approximately 54.5% of pediatric patients admitted to PICUs are unable to mobilize during their stay, underscoring the need for interventions to prevent immobility-related functional decline [20]. Early mobilization has shown effectiveness in adults by reducing functional deterioration, the duration of mechanical ventilation, and hospital length of stay [21,22]. In pediatric populations, it is considered a feasible and safe intervention when applied under conditions of ventilatory and hemodynamic stability [6,23]. However, the term lacks a standardized definition and includes a broad range of physical interventions, ranging from passive positioning to assisted ambulation and therapeutic play. In pediatrics, early mobilization refers to physical interventions initiated within the first 48 to 72 h of PICU admission to prevent complications associated with prolonged immobility (Figure 1) [3].

Based on the above, and with the aim of providing relevant information to facilitate the implementation of safe practices in PICUs and standardize knowledge of early mobilization in this population, the purpose of this review was to describe early mobilization protocols applied to critically ill pediatric patients in PICUs, analyzing the strategies employed, tools used, and barriers perceived by healthcare teams during implementation.

## 2. Materials and Methods

### 2.1. Study Design

This scoping review followed the guidelines established by PRISMA-ScR (Preferred Reporting Items for Systematic Reviews and Meta-Analyses Extension for Scoping Reviews) [24].

### 2.2. Search Strategy

#### 2.2.1. Search Sources

A systematic search was conducted across five electronic databases: PubMed, Scopus, Web of Science, Dimensions AI, and ScienceDirect. The final search update was performed in December 2024. 

#### 2.2.2. Review Question

The research question was structured using the PICO model: what are the early mobilization protocols used in critically ill pediatric patients in intensive care units, including the strategies employed and the barriers perceived by healthcare teams? This model has the following components:

▪ Population: Critically ill pediatric patients in intensive care units;
▪ Intervention: Early mobilization;
▪ Comparison: Not applicable;
▪ Outcomes: Identification of early mobilization protocols used, strategies implemented, and barriers perceived by healthcare teams in their implementation for this population.

### 2.3. Search Terms

The search query was developed using standardized terms from DeCS/MeSH and logical operators such as “OR” and “AND”. This methodology generated a search equation aligned with the review’s objectives: ((((((Early Ambulation) OR (Mobilization)) AND (Guidelines as Topic)) OR (Protocols)) AND (Pediatrics)) OR (Child) AND (Respiration Artificial)) AND (Hypotension). The search queries used in each of the five databases are summarized in Appendix A.

### 2.4. Eligibility Criteria

The inclusion criteria considered articles pertinent to the review topic, such as randomized clinical trials, observational studies, quasi-experimental studies, and protocols or guidelines addressing early mobilization in critically ill pediatric patients admitted to intensive care units for over 24 h. Eligible studies were those in which the methodology involved early mobilization interventions as part of the treatment or care provided to patients. Only studies published in English and focusing on patients aged 0 to 18 years were included. No restrictions were imposed regarding the publication date of the articles.

Studies were excluded if they did not specifically address early mobilization in critically ill pediatric patients in intensive care settings, if the methodology or study design was unclear or inadequate, or if they lacked sufficient data for analysis. Additionally, reviews, editorials, book chapters, conference abstracts, and case studies were not considered for inclusion.

### 2.5. Study Selection

All authors with expertise in the field contributed to data collection and management. Initially, a review of the titles and abstracts of the retrieved studies was conducted, and duplicates were removed using Mendeley 2021. Subsequently, a second review of the titles and abstracts was performed to identify studies for full-text reading, ensuring the inclusion of those meeting the predefined inclusion criteria. A detailed evaluation of the selected articles was then carried out, including only those that met the established eligibility criteria. In cases of disagreement regarding study selection, discrepancies were resolved through discussion, and when necessary, a third reviewer was consulted to reach a consistent and rigorous consensus in the selection process.

### 2.6. Quality Assessment of Studies

The methodological quality of the studies was evaluated according to their specific design. Observational studies, including cohort, cross-sectional, and longitudinal studies, were assessed using the Newcastle–Ottawa Scale [25], which evaluates three key domains: participant selection, group comparability, and outcome assessment. This scale allows for a detailed assessment of the risk of bias and internal validity.

Randomized controlled trials were evaluated using the Jadad Scale [26], which examines essential elements such as randomization, blinding, and dropout documentation. For non-randomized studies, the MINORS scale [27] was applied, which evaluates the clarity of objectives, the participant selection process, and the quality of outcome assessment, providing an integrated view of methodology in the absence of random control.

### 2.7. Data Extraction and Synthesis

Data extraction was performed independently for each selected study, thoroughly assessing the abstracts, methodologies, results, and conclusions. To synthesize the information, narrative methods were employed, and relevant articles were graphically represented using flowcharts. Relevant data were organized into tables to facilitate a detailed analysis of early mobilization protocols in critically ill pediatric patients in intensive care units. Additionally, prior systematic reviews were considered to provide supplementary context to the findings.

### 2.8. Transparency and Reproducibility

The process of data management and storage during the selection and extraction phases was thoroughly documented to ensure this study’s reproducibility.

## 3. Results

**Figure 2:**
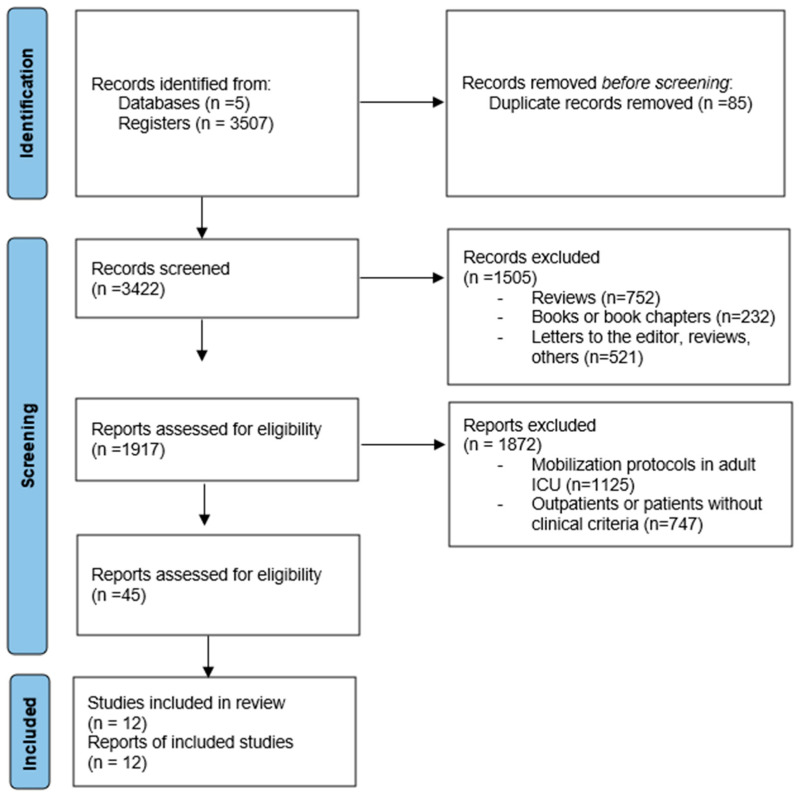
PRISMA flowchart for study selection.

A total of 3507 records were initially identified through searches in specialized databases. After removing duplicates, 3422 articles were screened. Following the application of inclusion and exclusion criteria, 46 articles were selected for full-text review. Ultimately, 12 studies were included in the final analysis, as they met the research objective and addressed the research question (Figure 2). These studies were assessed for methodological quality using the Newcastle–Ottawa [25], Jadad [26], and MINORS [27] scales. Among the selected studies, eight (72%) were observational studies, three (18%) were randomized controlled trials, and one (9%) was classified as non-randomized.

### 3.1. Methodological Quality of Studies

The evaluation of the methodological quality of the studies revealed that in the randomized controlled trials, 50% of the studies achieved the maximum score of 7/7 on the Jadad Scale [26], indicating excellent methodological quality, while the remaining 50% scored 4/7, reflecting acceptable methodological quality with some limitations when compared to the studies that achieved the maximum score (Appendix B.3).

In the observational studies, the only cohort study evaluated with the Newcastle–Ottawa Scale [25] obtained the highest score, indicating excellent methodological quality. Among the cross-sectional studies, 71.43% scored between eight and nine, which reflects good methodological quality (Appendix B.1 and Appendix B.2).

Finally, the only non-randomized study, evaluated using the MINORS scale [27], scored 20, reflecting its high methodological quality, with clear objectives, an appropriate participant selection process, and sufficient bias control (Appendix B.4).

### 3.2. Analysis of Study Design, PICU Stay, Measurements and Interventions

The included studies exhibited significant variability in their designs and methodologies, allowing for a detailed synthesis of the results. Table 1 outlines the characteristics of these studies, which encompass descriptive observational studies, both cross-sectional and longitudinal, such as those by Kudchadkar S et al. (2020) [28] and Wieczorek B et al. (2016) [29], conducted in the United States. These studies evaluated the prevalence and safety of rehabilitation in PICUs, respectively. Additionally, randomized controlled trials, including those by Fink E et al. (2019) [30] and Choong K et al. (2017) [27], investigated the feasibility and safety of protocolized rehabilitation and the use of bed ergometers as a complement to physiotherapy. Cohort studies, such as the one by Simpson C et al. (2022) [31] in Australia, and both prospective and retrospective studies, like those by Cui LR et al. (2017) [32], provided comprehensive insights into early mobilization interventions and their impact on PICU populations.

The studies present a wide range of designs, including descriptive observational studies, cohort studies, pilot trials, and randomized and non-randomized controlled clinical trials. All studies focused on critical illnesses requiring PICU admission with a minimum stay of 48 h, adhering to the established inclusion criteria. Interventions involved surveys, checklists, clinical data collection, forms, brochures, and physical therapy documentation, as well as the implementation of various protocols. These approaches evaluated the feasibility and safety of standardized physiotherapy for early mobilization in pediatric PICU patients.

Table 2 presents the early mobilization protocols used in the reviewed studies, identifying the types implemented and the responses observed in the pediatric population. The results provide a solid foundation for clinical applications in physiotherapy within the PICU setting. However, the lack of protocol standardization emphasizes the need for further research to optimize early mobilization practices.

### 3.3. Description and Analysis of Early Mobilization in PICUs

The reviewed studies report various early mobilization strategies implemented in Pediatric Intensive Care Units (PICUs), demonstrating feasibility and safety across different clinical settings. Wieczorek et al. (2016) [29] implemented an interdisciplinary protocol that included active in-bed mobility, ambulation, and staff education. This intervention was associated with a significant increase in mobilization events and physical therapy sessions starting from the third day of admission.

Choong et al. (2017) [33] integrated in-bed cycling into standard physical therapy, resulting in an increase in total mobilization time and higher physical activity intensity during the PICU stay. In another study, Abdulsatar et al. (2013) [34] employed interactive video gaming (Nintendo Wii™), specifically a boxing game, as a therapeutic tool. The intervention led to improvements in upper limb mobility, although no significant differences were observed in grip strength.

Colwell et al. (2018) [35] developed a multidisciplinary protocol that incorporated passive mobility, bed positioning, transfers, range of motion exercises, and ambulation. As a result, 52% of patients achieved the minimum mobilization targets defined by the protocol.

Collectively, these studies indicate that early mobilization in the PICU, when implemented through structured and interdisciplinary approaches, is feasible from the early stages of admission and is well tolerated by patients. Variations in intervention type reflect adaptability to clinical status and institutional resources.

## 4. Discussion

This systematic review assessed the impact of early mobilization in critically ill pediatric patients in PICUs, as well as the tools employed and the reported or perceived barriers to their implementation. We included 11 studies that demonstrated significant improvements, such as reductions in delirium and length of hospital stay. However, there remains a lack of specific data for this population. Early mobilization was shown to improve muscular functionality, physical performance, and post-discharge quality of life and reduce the duration of mechanical ventilation. Studies [28,29,30,31,32,37] highlight the importance of assessing hemodynamic stability, sedation levels, and pre-existing clinical conditions before initiating mobilization to mitigate associated risks.

Early mobilization was defined in the reviewed studies as any passive or active activity aimed at preserving or restoring musculoskeletal strength and function. In most of the analyzed protocols [28,29,30,31,32,36,37,38], mobilization began within the first 72 h after admission. Herbsman et al. (2020) [36] proposed a mobilization protocol within the first 18 h for non-ventilated patients and within 48 h for mechanically ventilated patients, optimizing adaptation to sedation and ventilatory support. These variations suggest that, despite the consensus on early mobilization’s benefits, there is no universal approach that can be adopted across all PICUs. Therefore, developing standardized protocols for different levels of severity and patient conditions remains a critical area for future research.

The studies emphasized the importance of evaluating hemodynamic stability, sedation levels, and pre-existing clinical conditions before initiating mobilization to reduce associated risks [28,29,30,31,32,37]. Functional and cognitive evaluations were measured in various studies using the POPC and PCPC, assessed both at admission and discharge. Specifically, Cui et al. (2017) [32] reported a 33% improvement in scores for these categories at discharge, while Fink et al. (2020) [30] used the FSS, along with the PRISM-III scale, to assess severity during the first 24 h of admission, providing a comprehensive analysis of patients’ functional progression and clinical condition.

The interventions included activities such as position changes in bed, passive exercises, rocking, and transfers and assisted ambulation outside the bed. In the “UCI Up” protocol by Wieczorek et al. (2016) [29], 27% of patients were able to ambulate by the third day, and 10% of mechanically ventilated patients were able to mobilize outside of the bed. Additionally, Ista et al. (2020) [37] observed that the likelihood of mobilizing out of bed was higher in non-ventilated patients. Kudchadkar et al. (2020) [28] documented that 30% of ventilated patients were able to mobilize out of bed, demonstrating that mobilization is feasible even with invasive ventilation. Azamfirei (2019) [39] noted that children admitted to PICUs during a critical period exhibit alteration in physical and neurocognitive development.

When comparing early mobilization to conventional management, Fink et al. (2020) [30] reported a higher frequency of out-of-bed activities and better functional outcomes (91% vs. 83%) in the intervention group. Ista et al. (2020) [37] pointed out that patients older than 3 years were more likely to ambulate easily, while over-sedation in younger patients limited mobilization. Kudchadkar et al. (2020) [28] also observed that family presence facilitated mobilization in 29% of patients under 3 years old.

Early mobilization has proven to be an effective strategy for managing critically ill pediatric patients, with significant benefits in terms of morbidity. However, studies such as Fink et al. (2020) [30] emphasize the need for further evidence to standardize protocols across PICUs. Research by Cui et al. (2017) [32] and Abdulsatara et al. (2013) [34,40] highlights the challenges in implementation due to varied clinical perceptions of its benefits and risks, underscoring the need for robust data to formalize its integration into PICU practices.

The safety of these interventions depends on the clinical and hemodynamic stability of the patient, particularly those on mechanical ventilation or under sedation. While passive mobilization techniques, such as postural changes and passive exercises, generally pose a low risk with proper monitoring, active mobilization, such as activities outside of bed, requires closer monitoring. Despite the low frequency of adverse events, such as oxygen desaturation and cardiovascular instability, the benefits of early mobilization—improving functionality and reducing the duration of mechanical ventilation—clearly outweigh the risks when protocols are followed correctly.

It is important to emphasize that the early mobilization process faces significant barriers that hinder its widespread adoption in PICUs. In the studies included in this review, the most frequently reported barriers were patient hemodynamic instability, excessive sedation, pain, insufficient staff, and lack of equipment availability. These findings are consistent with those reported in adult studies [41], which, while demonstrating that early mobilization is safe, feasible, and beneficial, also identify patient-related, structural, cultural, and process-related barriers that may limit the implementation of these practices. Therefore, it is essential to continue exploring strategies to successfully implement early mobilization in as many patients as possible, always ensuring patient safety.

The primary perceived and reported barrier to early mobilization is hemodynamic instability [41,42]. Although the literature has demonstrated its safety and feasibility in acute patients, healthcare providers often exhibit resistance to its execution in this patient group [43,44]. In this regard, interesting proposals have been made, such as those by Dubb et al. (2016) [41], who recommend a gradual mobilization approach, adhering to strict safety criteria, particularly in patients receiving vasopressors. Special attention is given to patients whose vasopressor doses have been increased, with recommendations to delay mobilization until at least two hours after dose adjustment.

Excessive sedation has also been identified in various previous studies [45,46] as a neuropsychological barrier to mobilizing PICU patients. To address this, some authors suggest implementing protocols aimed at achieving mild sedation levels, with routine sedation and pain assessments, following a transdisciplinary approach [41]. This approach seeks to provide opportunities for active or assisted mobilization whenever possible.

Also, the main structural or institutional barriers to early mobilization are the shortage of personnel and the lack of available equipment and supplies. This issue is well documented in the literature [47,48,49,50,51] and requires commitment and support from healthcare leadership and institutions, with a focus on increasing interdisciplinary staff and enhancing assistive technology. Recognizing the benefits and safety of these interventions, these steps are critical to ensuring the consistent implementation of early mobilization in PICUs worldwide.

In pediatric patients receiving Invasive Mechanical Ventilation, early mobilization carries several risks, the most significant of which is hemodynamic instability, which may manifest as fluctuations in blood pressure (>25%), hypotension, tachycardia, or arrhythmias, particularly in those requiring vasoactive agents. These changes can compromise the patient’s clinical stability, potentially necessitating the immediate cessation of the intervention or adjustments in mechanical ventilation parameters [6].

From a respiratory perspective, early mobilization may induce desaturation (SpO_2_ < 88%) or an increase in respiratory effort, especially in patients with recent tracheostomies or those dependent on high concentrations of oxygen [52,53].

An additional risk is the inadvertent manipulation of medical devices, such as endotracheal tubes, central catheters, or tracheostomy tubes, especially in younger or more agitated patients, which may compromise the effectiveness of ventilatory support and lead to additional complications [38,54]. Furthermore, early mobilization may interfere with continuous monitoring systems, such as oxygen saturation measurement and electrocardiography, hindering real-time assessment of physiological parameters and delaying the identification of potential adverse events [23,55].

### Limitations and Strengths

A notable limitation of this study is the heterogeneity of early mobilization protocols and the variability in patient clinical conditions, which complicate the direct comparison and generalization of results. The diversity in interventions applied and patient characteristics, such as the type of critical illness and pre-existing conditions, introduces variability that may affect the interpretation of early mobilization benefits. This underscores the need for standardized protocols and further studies with more homogeneous samples to facilitate a more accurate assessment of the effectiveness of these interventions. Additionally, the lack of pre- and post-intervention studies on early mobilization in pediatric patients with invasive ventilatory support limits the availability of robust evidence. The low prevalence of this practice further hinders the consistent collection of data necessary to assess its effectiveness and safety comprehensively.

A key strength of this review is its thorough analysis of the barriers perceived by healthcare teams, providing valuable insights into the factors that may affect the effective implementation of early mobilization protocols in pediatric critical care settings. This study highlights challenges related to clinical perceptions of risks and benefits, resource availability, and staff training—critical elements for the successful integration of these strategies into daily PICU practice. This focus helps to identify key areas that require attention in future research and clinical practice, promoting more effective and safer implementation of early mobilization in this vulnerable population.

## 5. Conclusions

Early mobilization in critically ill pediatric patients in PICUs demonstrates benefits such as improved mobility, reduced hospital stays, and decreased need for Invasive Mechanical Ventilation. However, the implementation of these activities faces barriers related to hemodynamic stability, use of sedation, and lack of personnel/tools. Interventions, including passive and active mobilization, typically begin within the first 24 to 72 h. Despite positive patient responses, further evidence is needed to establish uniform protocols and address current barriers, optimizing their effectiveness in this setting.

## Figures and Tables

**Table 1:** The characteristics of the included studies.

Author/Year	Kudchadkar, S, et al., 2020 [28]	Wieczorek, B, et al., 2016 [29]	Fink, E, et al., 2019 [30]	Simpson, C, et al., 2022 [31]	Cui, LR, et al., 2017 [32]	Choong, Karen MB, et al., 2017 [33]	Abdulsatar et al., 2013 [34]	Colwell BRL, et al., 2018 [35]	Herbsman, J, et al., 2020 [36]	Ista, E, et al., 2020 [37]	Simonassi, JI, et al., 2022 [38]
Objective	Analyze prevalence and barriers in pediatric rehabilitation	Evaluate safety and feasibility of early mobilization	Evaluate protocolized rehabilitation in neurocritically ill patients	Evaluate the impact of a checklist for early mobilization	Characterize the use of physical and occupational therapy in the PICU	Feasibility of bed cycling in the PICU	Evaluate the safety of virtual reality exercise in the PICU	Implement early mobilization protocol in the PICU	Increase early mobilization in orthopedic and neurosurgical patients	Prevalence and factors of physical rehabilitation in PICUs in Europe	Mobilization in the PICU with ventilatory support in a Latin American hospital
Sample	3098	200	110	71	138	30	8	567	403	456	196
Country	USA	USA	USA	Australia	USA	Canada	Canada	USA	USA	Europe	Argentina
Study design	Observational, cross-sectional	Observational, longitudinal	Randomized controlled trial	Cohort study	Observational, cross-sectional	Pilot randomized controlled trial	Pilot clinical trial	Observational cross-sectional	Observational longitudinal	Multicenter cross-Sectional	Observational, cross-sectional retrospective
Cause of PICU admission	Cardiac diseases	Critical illnesses	Traumatic brain injury, cardiac arrest, stroke	Critical illnesses	Pulmonary, gastrointestinal, neurological, transplant, cancer	Pulmonary, neurological, cardiac	Critical illnesses	Chronic diseases	Trauma, respiratory difficulty	Cardiorespiratory and post-surgical diseases	Acute respiratory infection
Age (years)	0–18	0–17	17–3	≥0.7–18	1 week–18 weeks	17–3	18–3	0–<4	≥18 months	0–<18	<18
PICU length of stay	≥72 h	≥72 h	≥48 h	>48 h	≥72 h	≥48 h	>48 h	Not available	≥48 h	≥72 h	>24 h
Interventions and duration	Mobilization in and out of bed, within the first 3 days after admission	PICU Up program: passive and active mobilizations, sleep hygiene, delirium control, within the first 3 days	PT, OT, SLT within the first 3 days after admission	Mobilization in and out of bed, within the first 2 days after admission	PT and OT Mobilization in and out of bed, within the first 3 days after admission	Bed cycling with ergometer + habitual physiotherapy, 210 min per week for 5 days	Virtual reality exercise (WiiMT), 10 min twice a day for 2 days	Mobilization in and out of bed, 2–3 times per day	Mobilization in and out of bed between 18 and 48 h after admission	Mobilization in and out of bed for patients admitted >72 h	Mobilization in and out of bed from 72 h after admission

PICU: Pediatric Intensive Care Unit; PT: physical therapy; OT: occupational therapy; SLT: Speech and Language Therapy; WiiMT: Wii Mobilization Therapy; USA: United States of America.

**Table 2:** Descriptions of early mobilization protocols in the PICU.

Author/ Year	Choong, Karen MB, et al., 2014 [1]	Kudchadkar, S, et al., 2020 [28]	Wieczorek, B, et al., 2016 [29]	Fink, E, et al., 2020 [30]	Simpson, C, et al., 2022 [31]	Cui, LR, et al., 2017 [32]	Choong, K, et al., 2017 [33]	Abdulsatara, F, et al., 2013 [34]	Colwell, B, et al., 2018 [35]	Herbsman, J, et al., 2020 [36]	Ista E, et al., 2020 [37]	Simonassi JI, et al., 2022 [38]
Measurement Scales	PCPC, POPC	PCPC	PICU Up! Questionnaire	FSS, POPC, PCPC	Early Mobilization Checklist	POPC	PEDI-CAT	Accelerometers, Grip-A.MT, PCPC, POPC	POPC	Algorithm: “ready for mobilization”	PCPC	POPC, CAP-D
Description of mobilization activities	Strengthening exercises, ambulation, transfers, chest physiotherapy	Passive mobility, sitting/standing, transfers, walking	Passive and active mobilization, positioning, ambulation, bed transfers	Positioning, passive/active range of motion, transfers, ambulation	In-bed mobility, edge of bed mobility, out-of-bed mobility, ambulation	Passive/active range of motion, transfers, resistance exercises, ambulation	In-bed cycling (30 min/day, 5 days/week), regular physiotherapy	WiiMT boxing exercises (10 min, twice a day for 2 days)	Passive and active movement, sitting, standing, transfers, ambulation	Active mobilization in bed, sitting on edge of bed, standing, ambulation	Passive range of motion, bed exercises, transfers, walking	Passive/active mobilization, transfers, ambulation, coordination exercises, strengthening
Contraindications for early mobilization	Excessive sedation, vasoactive infusions	Cardiovascular instability, excessive sedation	ECMO, open chest/abdomen, unstable fracture	Imminent death, PCPC 4-5	Not specified	Tachycardia, desaturation	Hemodynamic instability	Cardiopulmonary instability	Desaturation, tachypnea, emesis	Severe illness, incomplete data	Cardiac instability, sedation	Clinical severity, seizure disorders
% Ventilated	Not specified	59%	Not specified	74%	44%	65%	Not specified	50%	Not specified	13.11%	IMV 39.0%, NIMV 11.8%, MVTQT 12.9%	63.3% VMI, 37.7% VMNI
Perceived and declared barriers	Sedation, neuromuscular blockade, vasoactive infusions	Medical contraindications, hemodynamic instability, excessive sedation, lack of medical order	Medical procedures, hemodynamic instability, bed rest orders, equipment availability	Hemodynamic instability, abnormal intracranial pressure, parental/nursing refusal	Sedation, mechanical ventilation,	Hemodynamic instability, nursing request, patient absence	Availability of physiotherapist	Excessive sedation, ward transfers	Hemodynamic instability, lack of personnel	Lack of resources, equipment, lines/drains, patient agitation, confusion	Hemodynamic instability, excessive sedation	Not specified
Response to early mobilization	Improved peripheral and respiratory muscle strength and physical function and increased ventilator-free days	Safe mobilization; improved ambulation in children ≥ 3 years	Increased mobilization post-implementation	Functional improvement	Not applicable	Post-PICU functional improvement (28% ambulated)	Safe and feasible; enhanced intensity and duration of mobilization	Significant upper extremity activity increase	Increased mobilization; some ventilated patients able to walk	Early mobilization reduced hospital stay by 35%	Increased mobilization in children ≥ 3 years with family present	Early mobilization feasible in critically ill children on ventilatory support

PCPC: Pediatric Cerebral Performance Category; ECMO: Extracorporeal Membrane Oxygenation; POPC: Pediatric Overall Performance Category; CAP-D: Pediatric Delirium Assessment; FSS: Functional Status Scale; PEDI-CAT: Pediatric Evaluation of Disability Inventory-CAT; WiiMT: Wii Motion Therapy for Upper Extremities; IMV: Invasive Mechanical Ventilation; NIMV: Non-Invasive Mechanical Ventilation; MVTQT: Mechanical Ventilation with Tracheostomy.

## Abbreviations

The following abbreviations are used in this manuscript:

PICUs	Pediatric Intensive Care Units
ICUAW	Intensive Care Unit-acquired Weakness
NIMV	Non-Invasive Mechanical Ventilation
IMV	Invasive Mechanical Ventilation
MAP	Mean Arterial Pressure
PCPC	Pediatric Cerebral Performance Category
ECMO	Extracorporeal Membrane Oxygenation
POPC	Pediatric Overall Performance Category
CAP-D	Pediatric Delirium Assessment
FSS	Functional Status Scale
PEDI-CAT	Pediatric Evaluation of Disability Inventory-CAT
WiiMT	Wii Motion Therapy for Upper Extremities

## Appendix A. Database Search Queries

**Database**	**PubMed**	**Scopus**	**Web of Science**	**Dimensions AI**	**ScienceDirect**
Search Date	22 December 2024	22 December 2024	08 December 2024	14 December 2024	10 December 2024
Search Fields	All fields	All fields	All fields	Title and abstract	All fields
Search Equation	(((((Early Ambulation) OR (Mobilization)) AND (Pediatrics)) OR (Child)) AND (Respiration Artificial)) AND (Hypotension)	(((((Early Ambulation) OR (Mobilization)) AND (Pediatrics)) OR (Child)) AND (Respiration Artificial)) AND (Hypotension)	(((((Early Ambulation) OR (Mobilization)) AND (Pediatrics)) OR (Child)) AND (Respiration Artificial)) AND (Hypotension)	(((((Early Ambulation) OR (Mobilization)) AND (Pediatrics)) OR (Child)) AND (Respiration Artificial)) AND (Hypotension)	(((((Early Ambulation) OR (Mobilization)) AND (Pediatrics)) OR (Child)) AND (Respiration Artificial)) AND (Hypotension)
Records Identified	258	1.497	3	129	1620

## Appendix B

### Appendix B.1. The Newcastle–Ottawa Scale (NOS) for Assessing Study Quality—Cohorts

		**Selection**		**Comparability**		**Exposure**		**Score**
First Author & Reference	Representativeness of the Cohort Exposed	Selection of the Unexposed Cohort	Demonstration that the Outcome of Interest Was Not Present at the Start of the Study	Comparability of Cohorts Based on Design or Analysis	Evaluation of the Result	The Follow-Up Was Enough Prolonged for Them to Occur the Results?	Adequacy of Cohort Monitoring	Total
Choong, Karen MB, et al., 2014 [1]	*	*	*	*	*	*	*	7–9
Simpson C et al., 2022 [31]	*	*	*	**	*	*	*	8–9
Newcastle-Ottawa Scale (NOS) Criteria: Each star represents the achievement of a specific criterion on the scale. *: one criterion, **: two criteria.								

### Appendix B.2. The Newcastle–Ottawa Scale (NOS) for Assessing Study Quality—Cross-Sectional and Longitudinal Studies

**First Author & Reference**	**Kudchadkar, S, et al., 2020 [28]**	**Wieczorek, B, et al., 2016 [29]**	**Cui, LR, et al., 2017 [32]**	**Colwell B, et al., 2018 [35]**	**Herbsman, J, et al., 2020 [36]**	**Ista E, et al., 2020 [37]**	**Simonassi JI, et al., 2022 [38]**
Are eligibility criteria specified?	*	*	*	*	*	*	*
Representativeness of the sample	*	*	*	-	*	*	*
Sample selection/sample size	*	*	-	-	*	*	*
Definition of subjects not included	-	*	-	*	*	*	*
Comparability between participants	**	**	**	**	**	**	**
Outcome assessment	*	*	*	*	*	*	*
Same method of outcome assessment for the entire sample	*	*	*	*	*	*	*
Statistical test	*	*	*	*	*	*	*
Quantitative	9-8	9-9	9-7	9-7	9-9	9-9	9-9
Newcastle-Ottawa Scale (NOS) Criteria: Each star represents the achievement of a specific criterion on the scale. *: one criterion, **: two criteria							

### Appendix B.3. The Jadad Scale for Assessing the Quality of Randomized Clinical Trials

**First Author and Reference**	**Fink, E, et al., 2019 [30]**	**Choong, K, et al., 2017 [33]**
Is the study described as randomized?	1	1
Is the method used for random sequence generation described?	1	1
Is the method for generating the random sequence adequate?	1	1
Is the study described as double-blind?	1	0
Is the blinding method described?	1	0
Is the blinding method adequate?	1	0
Are the losses and withdrawals from the study described?	1	1
Quantitative score	7	4
Qualitative score	Good quality	Good quality

### Appendix B.4. The MINORS Scale for Assessing the Quality of Non-Randomized Studies

**First Author and Reference**	**Abdulsatara, F, et al., 2013 [34]**
Established objective	
Inclusion of patients consecutively	
Prospective data collection	
Appropriate data collection according to study objectives	
Unbiased outcome assessment	
Appropriate follow-up period according to study objectives	
Follow-up loss of less than 5%	
Sample size calculation (95% CI)	
Adequate control group	
Control and study groups managed simultaneously	
Baseline equivalence of groups	
Adequate statistical analysis	
Quantitative score	20
Qualitative score	Good quality
Green: 2 points; Yellow: 1 point; Red: 0 points.	
